# Influence of Al_2_O_3_ Additive on the Synthesis Kinetics of 1.13 nm Tobermorite, and Its Crystallinity and Morphology

**DOI:** 10.3390/ma18133086

**Published:** 2025-06-29

**Authors:** Raimundas Siauciunas, Liveta Steponaityte, Marius Dzvinka, Aivaras Kareiva

**Affiliations:** 1 Department of Silicate Technology, Kaunas University of Technology, Radvilenu 19, LT-50254 Kaunas, Lithuania; liveta.steponaityte@ktu.lt; 2 Faculty of Chemistry and Geosciences, Vilnius University, Naugarduko 24, LT-03225 Vilnius, Lithuania; marius.dzvinka@chgf.vu.lt (M.D.); aivaras.kareiva@chgf.vu.lt (A.K.)

**Keywords:** hydrothermal synthesis, Al-substituted tobermorite, C-S-H(I), properties of 1.13 nm tobermorite

## Abstract

One of the effective types of heat-resistant insulating products with an operating temperature of 1050 °C is made from calcium silicates or their hydrates. These materials are made from synthetic xonotlite and 1.13 nm tobermorite. Various wastes and by-products from other industries can be used for the synthesis of the latter compound. However, such raw materials often contain various impurities, especially Al-containing compounds, which strongly influence the kinetics of 1.13 nm tobermorite formation and its properties. Using XRD, DSC, TG, and SEM/EDX methods, it was found that at the beginning of the hydrothermal synthesis, the Al_2_O_3_ additive promotes the formation of 1.13 nm tobermorite; however, it later begins to inhibit the recrystallization of semi-crystalline C-S-H(I)-type calcium silicate hydrate and pure, high-crystallinity 1.13 nm tobermorite is more easily formed in mixtures without the aluminum additive. Aluminum oxide also influence the morphology of 1.13 nm tobermorite. When hydrothermally curing the CaO–SiO_2_ mixture, long, thin fibers (needles) are formed within 24 h. Later, they thicken and form rectangular parallelepiped crystals. After adding alumina, the product produced by 24 h synthesis is dominated by agglomerates, the surface of which is partially covered with crystal plates. By extending the synthesis duration, amorphous aggregates are absent and the crystal shape becomes increasingly square.

## 1. Introduction

The main measure to reduce heat loss is the use of effective insulation materials. Cellulose is environmentally friendly [1], but can only be used at temperatures of up to 550 °C and can cause allergic diseases [2]. So, in an environment with a temperature as high as 500 °C, silicate and ceramic materials should be used [3]. Glass fibers [4] can cause damage to the eyes, lungs, and even skin [5]. A good alternative is the use of inorganic fibers, which can be produced using waste and thus reduce the consumption of natural raw materials. Unfortunately, due to the high processing temperature, their production is associated with high energy consumption and CO_2_ emissions [6]. Another alternative is rock wool, but when used above temperatures of 400 °C, the fibers need to be bound with expensive binders [7,8]. Today, the most effective thermal insulating material is silica aerogel [9,10,11]. It is resistant to temperatures of up to 1100 °C and has a thermal resistance that is approximately two times higher than other similar products. Unfortunately, silica aerogels are expensive.

One of the effective varieties of heat-resistant insulating products with an operating temperature of 1050 °C is made from anhydrous calcium silicates or their hydrates [12,13,14]. Their properties are regulated by the standard EN 16977:2020 [15]. Calcium silicate hydrates (CSHs) can be synthesized from widely available raw materials, as well as from industrial by-products [16,17,18]. The most important performance characteristics are as follows: low density and thermal conductivity; high mechanical strength; good heat resistance; low shrinkage up to 1050 °C; durability and resistance to chemical corrosion; and ecological sustainability [19,20]. Due to their high specific surface area, calcium silicate hydrates can be used for hydrogen catalysis [21]. Their thermal stability depends on the temperature of recrystallisation to wollastonite (800–950 °C) [22], the presence of impurities, and the amount of formed intermediate amorphous or semi-crystalline phases [23].

From CSHs, xonotlite and 1.13 nm tobermorite Ca_5_Si_6_O_16_(OH)_2_·4H_2_O are used as thermal insulation materials. The latter compound forms in autoclaved aerated concrete, silicate bricks, CEDRAL slates, and insulation products. In addition, due to its unique crystal morphology (plates intertwined with fibers and nano dimensions), 1.13 nm tobermorite is used for the sorption of heavy and radioactive metal ions [24], in semiconductor production, and in medicine [25]. It is stable in mixtures with a CaO/SiO_2_ molar ratio of 0.70–0.83 and forms under hydrothermal conditions at 180–200 °C [26]. The properties of products made from xonotlite and 1.13 nm tobermorite are similar, but the latter has a lower operating temperature of 650–700 °C.

The formation of 1.13 nm tobermorite is a multi-stage process, as other calcium silicate hydrates may be formed at the beginning [27]. The purity of the resulting product depends largely on the parameters of the hydrothermal treatment [28]. Thus, the two main parameters that need to be considered are the quality of the raw materials and the synthesis mode [29]. When selecting raw materials for tobermorite synthesis, it is necessary to assess their availability, cost, and environmental impact. Various wastes and by-products from other industries are increasingly being chosen [12,30,31]. The advantages include reduced CO_2_ emissions, reduced waste in landfills, and the conservation of natural raw materials [32]. The most widely used by-products are microsilica [33], various slags [34,35], papermaking wastes [36], cullet [37], anhydrous silicates [38], and others.

Tobermorite, depending on the water content and the calcium–silicon ratio, is divided into several varieties: 0.93, 1.13, and 0.14 nm [39]. The 1.13 nm tobermorite is distinguished by its unique structural features and wide range of applications. Calcium ions and silicate chains are arranged in layers with a distance of ~1.13 nm between them. It is known that this mineral has two morphologies: needle-like crystals that are 1–2 µm long and plates that are 2–4 µm in size [39]. The synthesis process and properties of 1.13 nm tobermorite are greatly influenced by the introduction of foreign ions. The effect of aluminum-containing compounds has been studied most extensively [28,40]. Aluminum ions, when introduced into the tobermorite structure, change its crystal structure, morphology, chemical, and physical properties. Lothenbach et al. found that all Al^3+^ ions are located in the silicate chain, in the most protruding places, connected to two paired SiO_4_^4−^ tetrahedra and also to SiO_2_ in the adjacent silicate chain. It was also observed that Al^3+^ ions slightly reduce the degree of polymerization of 1.13 nm tobermorite [41].

Calcium silicate hydrate heat-resistant materials today constitute a separate class. The world’s largest manufacturers (CCEWOOL, Thermomax Inc., YUFENS) offer around 100 different products. They are used in metallurgy; combustion and annealing furnaces; gas and hot air ducts; the petrochemical, cement, and glass industries; power plants; domestic fireplaces; and many other areas. The development of the industry is also fueled by intensive research, with several hundred articles being published on the subject every year.

However, we believe a few fundamental issues remain unaddressed:

The literature indicates that 1.13 nm tobermorite crystals can be both plate-like or needle-like fibers. However, there are no detailed studies on when the crystals of the respective morphology predominate. Solving this problem and shifting the synthesis process towards the formation of a fibrous structure would eliminate the need for the addition of reinforcing additives to the product formation mix, leading to an internal reinforcement phenomenon.

Al^3+^ ions intercalated into the crystal lattice of 1.13 nm tobermorite significantly increase the shrinkage before it recrystallizes into wollastonite. This results in an ~100 °C lower operating temperature.

The 1.13 nm tobermorite is formed through intermediate, semi-amorphous C-S-H(I)-type compounds. Their thermal shrinkage is high [42]. For this reason, compounds suitable to produce heat-resistant materials must be synthesized at ~200 °C for 16–24 h.

The aim of this study is to examine some aspects of the influence of alumina on the hydrothermal synthesis process of 1.13 nm tobermorite, and its properties and morphology, as well as the sequence of formation of calcium silicate hydrate phases.

## 2. Materials and Methods

### 2.1. Materials and Raw Mixture Preparation

In this study, the materials used as the source of silica were amorphous SiO_2_·nH_2_O (reagent, 99.8%, Reachim, Moscow, Russia, analytical grade, loss on ignition: 18.32%) and microsilica (Mikrosill Gray, RUFAX, Kaunas, Lithuania). The X-ray analysis shows no diffraction peaks, only a saddle, which indicates that microsilica is an amorphous material. The chemical composition (determined with an X-ray fluorescence analysis) is presented in Table 1.

Sources of amorphous SiO_2_ were milled in a vibrating planetary mill Fritsch Pulverisette 9 (Fritsch GmbH, Idar-Oberstein, Germany) for 2 min at 900 rpm. The specific surface area of SiO_2_·nH_2_O–S_a_ is ~1000 m^2^·kg^−1^ and of microsilica S_a_ = 920 m^2^·kg^−1^.

CaCO_3_ (Stanchem, Niemce, Poland, analytical grade) was calcined at 950 °C for 1 h and milled in a vibrating planetary mill until reaching S_a_ = 620 m^2^·kg^−1^. The amount of free CaO was equal to 99.5%.

Al(OH)_3_ was purchased from Sigma-Aldrich (Hamburg, Germany, analytical grade). Amorphous Al_2_O_3_ was obtained by burning aluminum hydroxide at a temperature of 475 °C for 5 h (S_a_ = 750 m^2^·kg^−1^).

The mixtures, whose composition corresponds to the molar rations CaO/(SiO_2_ + Al_2_O_3_) = 0.83 and Al_2_O_3_/(SiO_2_ + Al_2_O_3_) = 0–0.075 (abbreviations: C/(S + A) and A/(S + A)), were prepared in a Turbula Type T2F homogenizer (Willy A Bachofen AG, Muttenz, Switzerland) for 45 min at 49 rpm.

**Figure 1:**
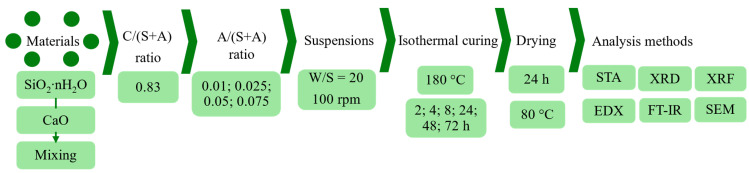
Scheme of the stages of experimental work.

Hydrothermal syntheses were carried out in stirred suspensions (100 rpm) in a 600 mL autoclave (Parr Instruments, model 4751, Moline, IL, USA) at 180 °C for 2–72 h (the water/solid ratio was equal to 20). The products were filtered, rinsed with isopropanol to reduce carbonization, dried at a temperature of 80 ± 1 °C for 24 h, and passed through a sieve with a 100 μm mesh. The consistent sequence of experimental work is presented in Figure 1.

### 2.2. Instrumental Analysis

The chemical composition analysis of the microsilica was performed by X-ray fluorescence spectroscopy (XRF) on a Bruker X-ray S8 Tiger WD spectrometer (Bruker AXS GmbH, Karlsruhe, Germany). The data were analysed with SPECTRA Plus QUANT EXPRESS (Poulsbo, WA, USA) standardless software.

X-ray diffraction (XRD) patterns were recorded on D8 Advance instrument (Bruker AXS GmbH, Karlsruhe, Germany). The main measurement parameters, determination, and calculation methodology are presented in reference [27]. The software used for compound identification was Diffrac.eva v3.0 (Bruker AXS GmbH, Karlsruhe, Germany).

A thermal analyzer (Linseis PT1000, Linseis Massgeraete GmbH, Selb, Germany) was applied to simultaneous thermal analysis (STA: differential scanning calorimetry (DSC) and thermogravimetric (TG)) studies. Heating was carried out under a N_2_ atmosphere at a heating rate of 10 °C·min^−1^; the temperature ranged from 30 up to 1000 °C. Ceramic sample handlers and crucibles of Pt were used. The mass of all tested samples was 15 ± 0.3 mg.

The surface structure of the samples was investigated using a Hitachi SU-70 (Chiyoda, Japan) scanning electron microscope. To increase the surface electrical conductivity, the samples were coated with a 15 nm layer of silver. Elemental composition was obtained using the Hitachi FlexSEM 1000 II scanning electron microscope with an Oxford Instruments energy-dispersive X-ray spectrometer (Quorum Technologies Ltd., Laughton, United Kingdom; AztecOne software). For these measurements an accelerating voltage of 15 kV was use.

The specific surface area was determined by using the CILAS LD 1090 (Cilas, Orléans, France, dry method, air supply—6 bar, Toni Technik 7201, Berlin, Germany).

The density was determined by the Ultrapyc 1200e (Micromeritics, Norcross, GA, USA).

CaCO_3_ was burned and losses on ignition (LOI) were determined in the Nabertherm LV 15/11/P330 (Bremen, Germany).

## 3. Results

### 3.1. Influence of Al_2_O_3_ on the Mineralogical Composition of Synthesis Products

**Figure 2:**
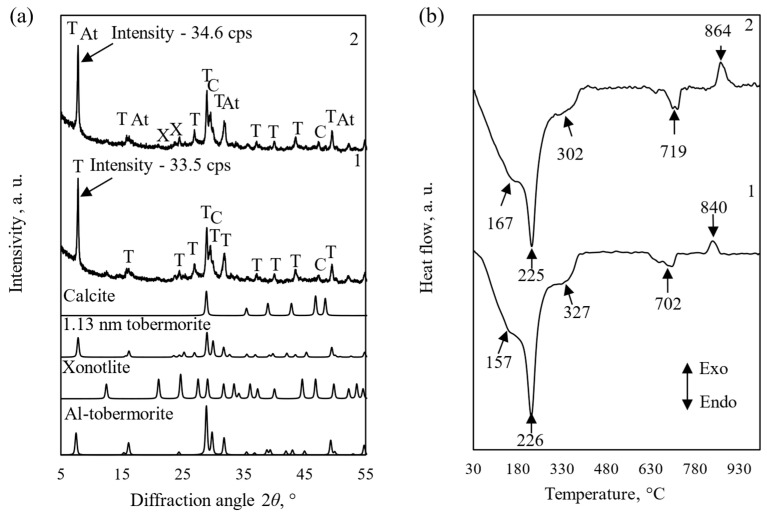
XRD patterns (**a**) and DSC curves (**b**) of synthesis products. The 1–72 h synthesis when CaO/SiO_2_ = 0,83; this product + 5% Al_2_O_3_, after 24 h. Indexes: T—1.13 tobermorite; C—calcite, At—Al-substituted tobermorite, X—xonotlite.

It is well known that pure 1.13 tobermorite in the DSC curve is characterized only by an endothermic effect at a temperature of ~250 °C (the compound loses four molecules of crystal water) [43], while C-S-H(I)-type semi-amorphous calcium silicate hydrate is characterized by an exothermic effect at a temperature of 820–850 °C (recrystallization into wollastonite) [44]. However, it is often stated in the scientific literature that Al-substituted tobermorite is characterized by both transformations—at temperatures of ~250 °C and 820–850 °C [40]. In our opinion, this is an incorrect interpretation of the data. To support this position, we synthesized practically pure 1.13 nm tobermorite from a mixture of CaO and SiO_2_ reagents (CaO/SiO_2_ = 0.83) in a hydrothermal environment at 180 °C within 72 h (Figure 2a, curve 1; *d*-spacing = 1.130, 0.308, 0.298, 0.184, 0.0282 nm, PDF 00-019-1364). The obtained DSC results (Figure 2b, curve 1) are also in good agreement with the literature data. The endothermic peak at ~225 °C is attributed to the dehydration of 1.13 nm tobermorite. The endothermic effect in the temperature range of 700–720 °C is caused by the decomposition of calcite. It is doubled, which indicates that the synthesized products contain not only crystalline but also amorphous CaCO_3_ [45]. The latter was most likely formed during the filtration and drying of the samples. The exothermic effect at 840 °C shows that the semi-crystalline C-S-H(I)-type calcium silicate hydrate recrystallizes into wollastonite.

We mixed synthetic 1.13 nm tobermorite with 5% Al_2_O_3_ and repeatedly synthesized at 180 °C for 2–24 h. We determined using XRD and DSC methods that no other aluminum-containing compounds were formed (Figure 2, curves 2). In addition, the product’s *d*-spacing values change, albeit slightly (Table 2). The ionic radius of Al^3+^ is 0.050 nm or 50 picometres. The ionic radius of Si^4+^ is 0.041 nm (41 picometers) when it is in a four-coordinate, tetrahedral environment. Thus, when an element with a larger ionic radius is introduced into the 1.13 nm tobermorite crystal lattice (most likely by replacing Si^4+^), the distance between the planes in it increases slightly.

This means that the entire amount of Al_2_O_3_ added is intercalated into the 1.13 nm tobermorite structure, but the area of the exothermic peak characteristic of the recrystallization of semicrystalline C-S-H(I) into wollastonite remains almost unchanged. The main differences observed were that very little xonotlite was formed (*d*-spacing = 0.361, 0.422 nm, PDF 00-002-0355), and the exothermic effect shifted by ~20 °C to a higher temperature. For this reason, we identified the products with peaks characteristic of 1.13 tobermorite in the XRD patterns and thermal effects at ~250 °C and 820–870 °C in the DSC curves as a mixture of 1.13 nm tobermorite and C-S-H(I).

**Figure 3:**
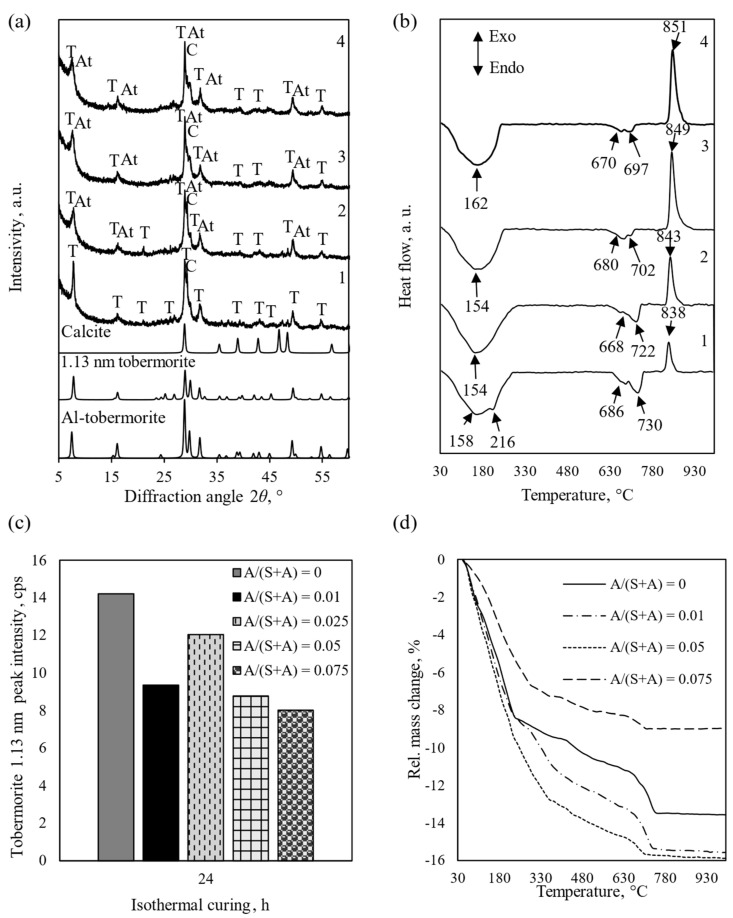
XRD patterns (**a**), DSC curves (**b**), tobermorite 1.13 nm peak intensity (**c**), and TG curves (**d**) of the synthesis products, when 1—A/(S + A) = 0; 2—A/(S + A) = 0.01; 3—A/(S + A) = 0.05; 4—A/(S + A) = 0.075. Indexes: T—1.13 nm tobermorite; C—calcite, At—Al-substituted tobermorite.

First, the dependence of the kinetics of calcium silicate hydrate formation on the amount of added Al_2_O_3_ was investigated. The molar ratio A/(S + A) varied from 0.01 to 0.075, and the synthesis time was 24 h (selected according to the data from the literature source [18]. The data are presented in Figure 3.

XRD analysis shows that in the mixture without the addition of Al_2_O_3_, a sufficiently large amount of 1.13 nm tobermorite is formed under these conditions (Figure 3a, pattern 1). It is worth noting that the DSC curve already shows the dehydration effect characteristic of 1.13 nm tobermorite at a temperature of 216 °C (Figure 3b, curve 1), while after the addition of Al_2_O_3_, we see only a wide, diffused bend characteristic of the dehydration of semi-amorphous C-S-H(I) (Figure 3b, curves 2–4). The more Al_2_O_3_ is added to the mixture, the more the degree of crystallinity of the products decreases (Figure 3a), and the heat flux released during the recrystallization of C-S-H(I) into wollastonite increases (when A/(S + A) = 0.01–10 mW, when A/(S + A) = 0.05–16 mW, when A/(S + A) = 0.75–18 mW), and the point of reaction shifts towards higher temperatures (Figure 2b; 840–850 °C). They also contain Al-substituted tobermorite (*d*-spacing = 0.3090, 1.1800, 0.2995 nm, PDF 00-019-0052) and calcite formed during filtration and drying of the suspension (*d*-spacing = 0.3042, 0.1879, 0.2289 nm, PDF 01-078-3262). TG analysis showed that most CaCO_3_ is formed in the mixture without Al_2_O_3_ or with a small amount of it. When A/(A + S) = 0; 0.01; and 0.05, the total mass losses are 13.6, 15.6 and 15.9%, respectively. When the molar ratio is increased to A/(A + S) = 0.075, the mass losses decrease to 9%. The reason for this phenomenon is unclear to us and requires further investigation.

**Figure 4:**
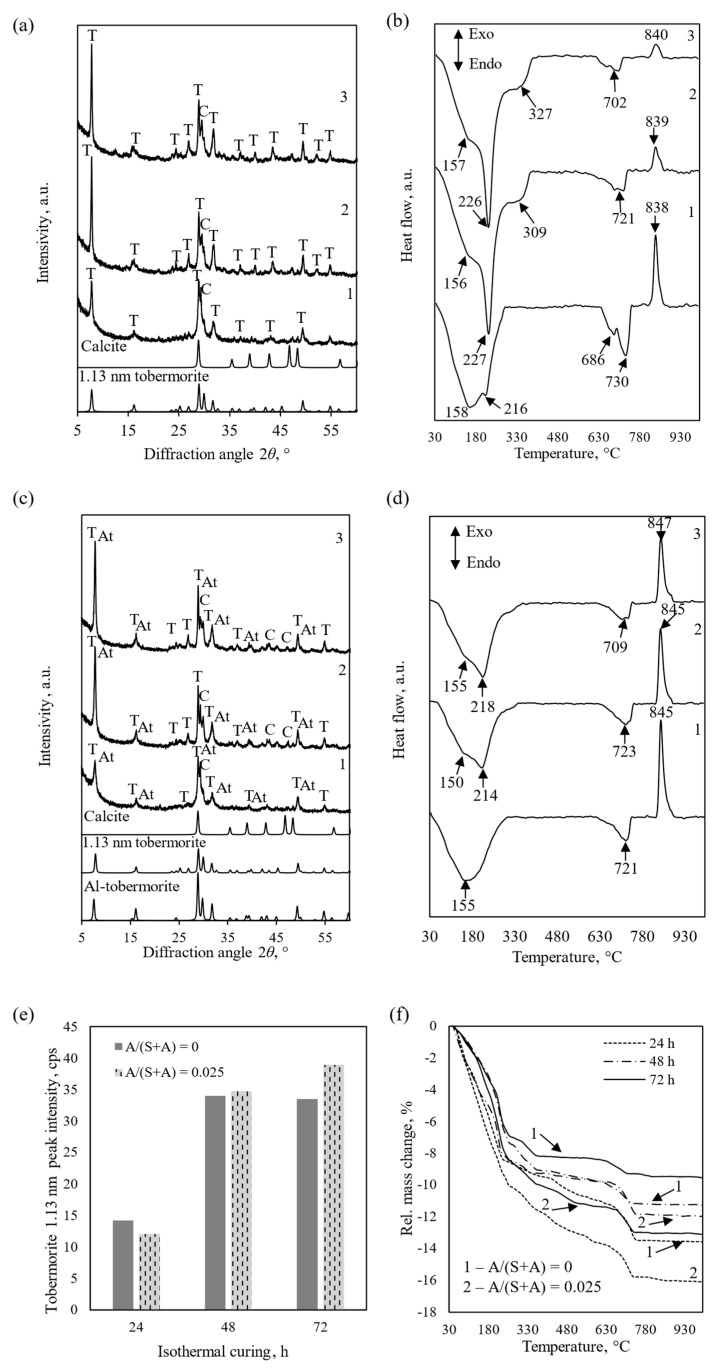
XRD patterns (**a**,**c**), DSC curves (**b**,**d**), tobermorite 1.13 nm peak intensity (**e**), and TG curves (**f**) of the synthesis products, when A/(S + A) = 0 (**a**,**b**) and 0.025 (**c**,**d**). Duration of syntheses: 1—24 h; 2—48 h; 3—72 h. Indexes: T—1.13 nm tobermorite; C—calcite, At—Al-substituted tobermorite.

For the above reasons and due to the scope of this study, all further studies were carried out using a mixture with molar ratios of C/(S + A) = 0.83 and A/(S + A) = 0.025. First, the influence of the duration of hydrothermal synthesis on the phase composition of the resulting products was investigated. The data are presented in Table 3 and Figure 4. As we can see from the results in Table 3, the heat flux released by the exothermic transformation of the product obtained in the mixture without Al_2_O_3_ (2 h synthesis) at a temperature of ~840 °C (C-S-H(I) transition to wollastonite) is 37 mW, and with alumina, it is almost twice as low. This, perhaps not entirely directly, indicates that at the beginning of the hydrothermal synthesis, the Al_2_O_3_ additive promote the formation of 1.13 nm tobermorite. By extending the synthesis time to 4–8 h, the values of the heat flux released during the recrystallization into wollastonite of the products obtained from both mixtures are close. By increasing the duration of the hydrothermal treatment to 24–72 h, the values of this flux in the product made from the pure mixture consistently decrease and approach nearly zero, while in the mixture with the Al_2_O_3_ additive, a sufficiently intense effect remains in the DSC curve at a temperature of ~845 °C (Figure 4d).

Thus, at the beginning of the hydrothermal synthesis, the Al_2_O_3_ additive promotes the formation of 1.13 nm tobermorite. We assume that the main reason is as follows: The solubility of amorphous SiO_2_ at room temperature is much higher than that of CaO. With increasing temperature, the solubility of silica increases further, while that of CaO decreases. For this reason, the CaO/SiO_2_ molar ratio in the reaction medium is much lower than the stoichiometric ratio of 1.13 nm tobermorite (0.83). It is known [46] that even small amounts of Al^3+^ ions reduce the solubility of SiO_2_ by tens of times. As a result, the CaO/SiO_2_ ratio in the reacting medium approaches the optimal ratio and favorable conditions are created for the crystallization of 1.13 nm tobermorite. However, when the duration of hydrothermal synthesis is extended even to 72 h, in mixtures with an aluminum additive, a high-intensity exothermic effect at ~845 °C remains (Figure 4d, curve 3). In our opinion, this means that aluminum was incorporated not only in the 1.13 nm tobermorite crystal lattice but also in the C–S–H(I) structure and stabilized both compounds. It has long been and reliably been proven that Al^3+^ ions prevent the recrystallization of 1.13 nm tobermorite into xonotlite [26,27]. We believe that they analogously stabilize C-S-H(I) and prevent its transition to 1.13 tobermorite. Thus, if for practical purposes the product produced does not require high thermal stability and low shrinkage during combustion, it is worth using Al-containing additives—this reduces the synthesis temperature and duration, and reduces the cost of production. However, in cases where it is necessary to avoid the formation of a semi-amorphous phase, it is better to use raw materials containing only a minimal amount of impurities with Al-compounds.

**Figure 5:**
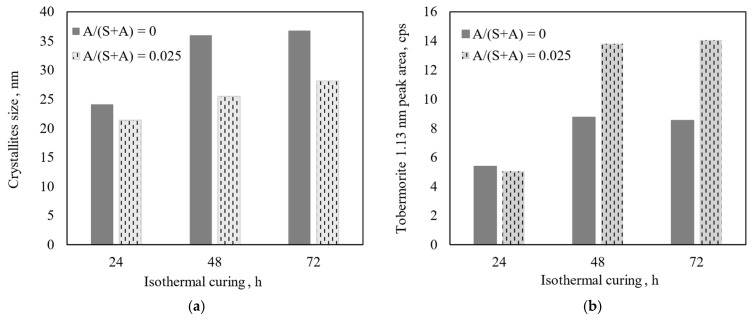
Dependence of the size of 1.13 nm tobermorite crystallites (**a**) and peak (*d*–spacing = 1.13 nm) area (**b**) on synthesis time.

The Al_2_O_3_ additive also affects the size and peak area of 1.13 nm tobermorite crystallites (Figure 5).

It was determined that the size of 1.13 nm tobermorite crystallites after 24 h of synthesis from the pure CaO–SiO_2_ mixture is larger than in the product with Al_2_O_3_ addition—24.1 and 21.4 nm, respectively (Figure 4a). The obtained results indirectly indicate that the formation of 1.13 nm tobermorite in a CaO–SiO_2_ mixture was accelerated significantly, changing only the synthesis duration—the size of the crystallites increases to 36 nm after 48 h. Comparing the data in Figure 4a,b and Figure 5a, it can be stated that 48 h is the optimal duration for the formation of 1.13 nm tobermorite with a high degree of crystallinity—when it was extended to 72 h, no significant differences were observed. Meanwhile, the crystallite size of Al-substituted tobermorite increased consistently, but very slowly, throughout the entire synthesis period and was significantly smaller than the crystallite size of pure 1.13 nm tobermorite (Figure 5a). The obtained data perfectly correspond to the Scherrer equation—the larger the crystallites (in this case, from the crystalline plane *h k l*; *d*–spacing 1.133 nm), the smaller the full width at half of the maximum intensity (Figure 5b).

### 3.2. Properties and Morphology of Pure and Al-Substituted Tobermorite

Hydrothermally synthesized 1.13 nm tobermorite with a high degree of crystallinity is most widely used today to produce low-density thermal insulation and heat-resistant (up to 750 °C) materials. The bulk density of the final product is controlled by regulating the proportion of water in the suspension, excluding the crystal water in calcium silicate hydrates. Physically bound water is usually removed during the formation of the products. Most commonly (in so-called filter press processes) some of the suspension medium is extruded from the reactive mixture during the formation process, when a sufficient reaction occurs to form a self-supporting but compressible gel. The slower the sedimentation rate of the synthesis suspension and the more physically bound water there is, the lower the bulk density of the products that can be formed [47]. Calcium silicate hydrates physically bind a large amount of water and acquire a consistency that is thicker than water. The relative volume of the synthesized product is estimated as the ratio of the suspension volume after 24 h of sedimentation to the initial mass of solids. The higher the relative volume, the lower the average density of the thermal insulation products that can be formed [42].

The data of the suspension’s relative volume and the granulometry of the synthesized products are presented in Table 4.

As we can see from the data presented, much finer particles are formed in a mixture with a molar ratio of A/(S + A) = 0, than of A/(S + A) = 0.025. The addition of Al_2_O_3_ increases the specific surface area by more than 1.5 times. Because the particles are smaller, their sedimentation rate is lower, and due to their larger specific surface area, they physically bind more water.

This once again confirms that when manufacturing products in which amorphous or semi-crystalline compounds negatively affect their performance properties, it is necessary to select raw materials with a minimum amount of Al-containing impurities.

**Figure 6:**
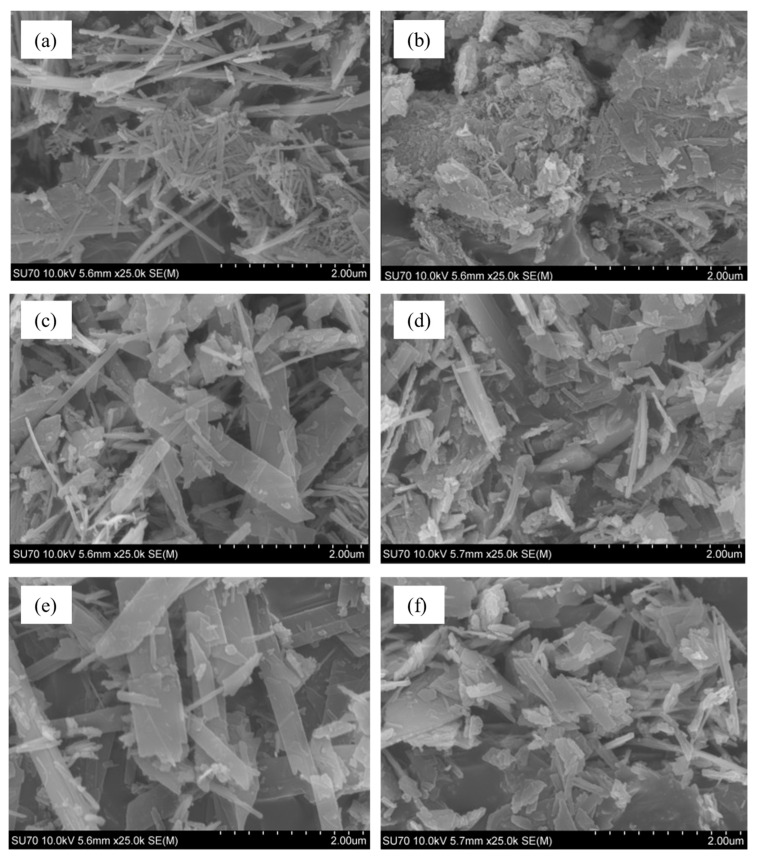
SEM images of syntheses products. Molar ratio of mixtures A/(S + A) = 0 (**a**,**c**,**e**) and A/(S + A) = 0.025 (**b**,**d**,**f**). Duration of hydrothermal syntheses at 180 °C—24 h (**a**,**b**), 48 h (**c**,**d**), and 72 h (**e**,**f**).

There is sufficient evidence in the scientific literature that 1.13 nm tobermorite can crystallize in both fibrous and plate-shaped crystals [35]. Unfortunately, the reasons for this unusual phenomenon are not entirely clear. Morphological studies of our synthesized products suggest that one of the reasons may be the presence of Al compounds in the reaction medium. Figure 6a clearly shows that the product synthesized within 24 h from the CaO–SiO_2_ mixture without the additive of Al_2_O_3_ is dominated by long (up to 12 nm), thin fibers. It is true that we also see an accumulation of small crystals, but it is also composed of fibers. After adding alumina to the initial mixture, the product of the 24 h synthesis is dominated by agglomerates (most likely semi-amorphous C-S-H(I)), the surface of which is partially covered with crystal plates (Figure 6b). This agrees well with the DSC data presented in Figure 4b,d, which shows that the heat flux value determining the transformation of C-S-H(I) to wollastonite in the product with A/(S + A) = 0.025 is significantly higher than in the product with A/(S + A) = 0. After extending the hydrothermal treatment time to 48 h (pure CaO–SiO_2_ mixture), the fiber-shaped crystals of 1.13 nm tobermorite become coarser and form rectangular parallelepiped crystals (Figure 6c). Their length changes little (remains ~12 nm), but their width increases significantly (up to ~2 nm). In products with the addition of Al_2_O_3_, amorphous aggregates are absent, and plate-shaped crystals with similar length and width dimensions predominate. After extending the duration of hydrothermal synthesis to 72 h, the morphology of the products does not change significantly—the rectangular parallelepiped crystals in mixtures without the alumina additive become coarser (Figure 6e), while in products with the additive, the crystal shape becomes increasingly square (Figure 6f).

**Figure 7:**
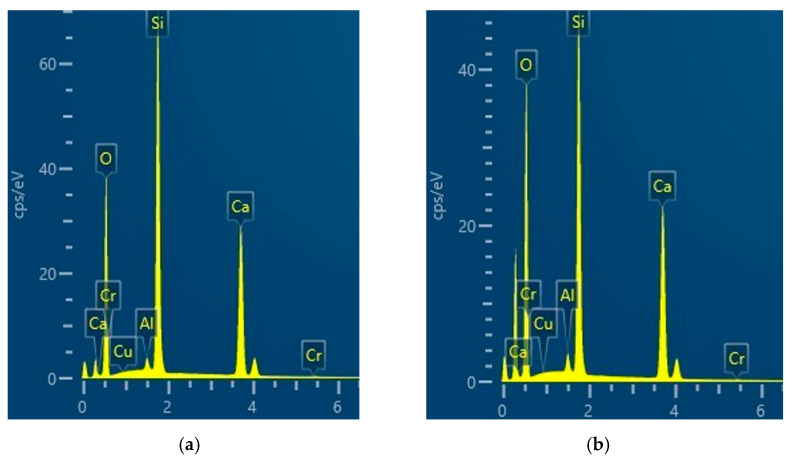
EDX data of 24 h (**a**) and 48 h (**b**) hydrothermal syntheses from the mixture with molar ratios A/(S + A) = 0.025 at 180 °C.

We determined the elemental composition of the resulting compounds using energy-dispersive X-ray spectroscopy. The goal was to ensure that all aluminum is intercalated into the 1.13 nm tobermorite crystal lattice structure. Measurements were performed on the surface of five different crystals and the results of these studies are presented in Figure 7, and Table 5 presents the data (average of five measurements), which were obtained by converting the elemental composition to the oxide composition, as well as the composition of the products in molar ratios. The composition of the initial mixture is also provided for comparison.

The obtained results only confirm the data previously published by other authors [48], in that Al^3+^ ions very easily intercalate into the 1.13 nm tobermorite crystal lattice structure. However, they were necessary for us to show that the changes in the mineralogical composition, properties, and morphology of synthesized products are determined precisely by the interference of aluminum, and not by other reasons. As we can see from the presented data, the composition of the synthesized Al-substituted tobermorite (regardless of whether 24 h or 48 h of hydrothermal treatment had been carried out at 180 °C) is very close to the composition of the initial mixture and varies only within the limits of error.

## 4. Conclusions

The formation mechanism of 1.13 nm tobermorite is a complex process and is highly dependent on the hydrothermal curing parameters, reactivity of the raw materials, and the nature and amount of impurities they contain. In order to use various wastes and by-products from other industries, it is necessary to first assess the influence of foreign ions present in them on the synthesis kinetics of 1.13 nm tobermorite. It is best to start the study with model systems and determine the individual influence of each impurity. Both natural raw materials and industrial waste very often contain Al-containing compounds.

In this study, some aspects of the influence of alumina on the synthesis process and properties of 1.13 tobermorite were investigated.

1. The Al_2_O_3_ additive first aids in the formation of 1.13 nm tobermorite during the hydrothermal synthesis, but later begins to inhibit the recrystallization of C-S-H(I), and pure, high crystallinity 1.13 nm tobermorite is more easily formed in mixtures without the alumina additive.
2. Additives containing aluminum ions influence the morphology of 1.13 nm tobermorite. The product synthesized within 24 h from the CaO–SiO_2_ mixture without Al_2_O_3_ is dominated by long (up to 12 nm), thin fibers. By prolonging the synthesis to 48–70 h, they take on the shape of rectangular parallelepiped crystals. When alumina is added, the morphology changes—after 24 h agglomerates dominate, the surface of which is partially covered with crystal plates. By extending the curing time, amorphous aggregates are absent and the crystal shape becomes increasingly square.

## Figures and Tables

**Table 1:** Oxide composition of microsilica.

Oxides	SiO_2_	CaO	Al_2_O_3_	K_2_O	Na_2_O	MgO	SO_3_	Other	LOI
**Content, %**	97.7	0.59	0.13	0.53	0.18	0.26	0.25	0.17	2.51

**Table 2:** The *d*-spacing and PDF number of 1.13 nm tobermorite and Al-substituted tobermorite.

Compound	*d*-Spacing, nm					PDF Number
1.13 nm tobermorite	1.130	0.3080	0.2980	0.1842	0.2820	00-019-1364
Al-substituted tobermorite	1.180	0.3090	0.2995	0.1848	0.2814	00-019-0052

**Table 3:** Dependence of the heat flow of the exothermic effect at 830–840 °C on the synthesis duration and Al_2_O_3_ content.

C/(S + A)	A/(S + A)	Duration of Hydrothermal Treatment, h					
		2	4	8	24	48	72
		Heat Flux of Exothermic Effect at 830–850 °C, mW/g·10^3^					
0.83	0	2.47	1.47	1.06	0.42	0.14	0.08
	0.025	1.32	1.31	1.20	0.92	0.74	0.67

**Table 4:** Relative volume of the suspension and the granulometry of the synthesized products.

Sample After 24 h Curing at 180 °C	Relative Volume, cm^3^/g	Diameter, µm, at			Mean Diameter, µm	Specific Surface Area, m^2^/kg
		10%	50%	90%		
A/(S + A) = 0	16.2 ± 0.9	0.94	9.88	26.86	12.20	652.7
A/(S + A) = 0.025	13.4 ± 1.2	2.60	13.66	35.99	16.97	401.0

**Table 5:** Oxide composition and molar ratio of synthesized Al-substituted tobermorite.

Product	Oxide Content, wt%			Molar Ratio	
	CaO	SiO_2_	Al_2_O_3_	C/(S + A)	A/(S + A)
**Initial mixture**	43.22 ± 0.05	54.40 ± 0.06	2.37 ± 0.02	0.83	0.025
After 24 h synthesis	45.83 ± 0.06	52.66 ± 0.06	2.31 ± 0.01	0.818	0.0243
After 48 h synthesis	45.04 ± 0.04	52.50 ± 0.07	2.42 ± 0.03	0.804	0.0255

## Data Availability

The original contributions presented in this study are included in the article. Further inquiries can be directed to the corresponding author.

## Abbreviations

The following abbreviations are used in this manuscript:

CSH	Calcium silicate hydrate
C-S-H(I)	Semi-crystalline calcium silicate hydrate with a variable CaO/SiO_2_ molar ratio from 0.7 to 1.1
LOI	Losses on ignition
XRD	X-Ray diffraction
DSC	Differential scanning calorimetry
TGA	Thermogravimetric analysis
SEM	Scanning electron microscopy
EDX	Energy dispersive X-ray
A/(S + A)	Al_2_O_3_/(SiO_2_ + Al_2_O_3_)
C/(S + A)	CaO/(SiO_2_ + Al_2_O_3_)

## References

[1] Guo W., Shun C., Liang F., Jin L., Ji C., Zhang P., Fei B. (2023). Ultra-light-weight, anti-flammable and water-proof cellulosic aerogels for thermal insulation applications. Int. J. Biol. Macromol..

[2] Popov T.A., Emberlin J., Church M.K., Aberg N., Josling P. (2019). Powder cellulose in allergic rhinitis management: Relevance of vitro findings to real-life safety. Int. Arch. Allergy Immunol..

[3] (2005). Engineering ToolBox, Insulation Materials—Temperature Ranges. https://www.engineeringtoolbox.com/insulation-temperatures-d_922.html.

[4] Mahltig B., Kyosev Y., Martynova E., Cebull H. (2019). Inorganic and composite fibers. Production, properties, and applications. Chapter 7—Glass Fibers. Inorganic and Composite Fibers: Production, Properties, and Applications.

[5] Eske J. How to Remove Fiberglass in Skin. Medical News Today. https://www.medicalnewstoday.com/articles/fiberglass-in-skin.

[6] Zhang J., Xu X., Cheng F., Ramakrishna S. (2022). Study Progress on Inorganic Fibers from Industry Solid Wastes and the Key Factors Determining Their Characteristics. Materials.

[7] Tingley D.D., Hathway A., Davison B., Allwood D. (2017). The environmental impact of phenolic foam insulation boards. Proc. Inst. Civil Eng. Constr. Mater..

[8] Ko H., Lee H.S., Lim H.M. (2020). Effects of additives in colloidal silica based inorganic-hybrid binder for mineral wool insulation boards. J. Asian Ceram. Soc..

[9] Niculescu A.-G., Tudorache D.-I., Bocioagă M., Mihaiescu D.E., Hadibarata T., Grumezescu A.M. (2024). An Updated Overview of Silica Aerogel-Based. Nanomaterials.

[10] Lin J., Li G., Liu W., Qiu R., Wei H., Zong K., Cai X. (2021). A review of recent progress on the silica aerogel monoliths: Synthesis, reinforcement, and applications. J. Mater. Sci..

[11] Lei J., Zheng S., Han Z., Niu Y., Pan D., Liu H., Liu C., Shen C. (2024). A Brief Review on the Preparation and Application of Silica Aerogel. Eng. Sci..

[12] Qi F., Zhu G., Chen Y., Zhu Y., Li S., Zhang J., Li H. (2024). Mechanism of One-Step Hydrothermal Process to Prepare Tobermorite Thermal Insulation Materials during Recovery of Silicon-Rich Lye. Ind. Eng. Chem. Res..

[13] Ogur E., Botti R., Bortolotti M., Colombo P., Vakifahmetoglu C. (2021). Synthesis and additive manufacturing of calcium silicate hydrate scaffolds. J. Mater. Res. Technol..

[14] Liu J., Pan X., Guo Y., Zou Z., Wang Z., Yu H. (2024). Crystallization mechanism and physical properties of xonotlite intensified by inorganic and organic additives based on direct hydrothermal synthesis. J. Non-Cryst. Solids.

[15] (2020). Thermal Insulation Products for Buildings—Factory Made Calcium silicate (CS) Products—Specification.

[16] Galvankova L., Masilko J., Solny T., Stepankova E. (2016). Tobermorite synthesis under hydrothermal conditions. Procedia Eng..

[17] Jimenez I., Perez G., Guerrero A., Ruiz B. (2017). Mineral phases synthesized by hydrothermal treatment from biomass ashes. Int. J. Miner. Process..

[18] Smalakys G., Siauciunas R. (2018). The synthesis of 1.13 nm tobermorite from carbonated opoka. J. Therm. Anal. Calorim..

[19] Liu F., Cao J.X., Zhu B. (2011). Effect of anion impurity on preparing xonotlite whiskers via hydrothermal synthesis. Adv. Mater. Res. Trans..

[20] Pugovkina Y., Kutugin V., Ostroumova A., Rymanova I. (2016). High temperature and heat insulated calcium silicate materials. Key Eng. Mater. Trans..

[21] Akbayrak S., Ozkar S. (2016). Inverse relation between the catalytic activity and catalyst concentration for the ruthenium(0) nanoparticles supported on xonotlite nanowire in hydrogen generation from the hydrolysis of sodium borohydride. J. Mol. Catal. A Chem..

[22] Shaw S., Henderson C.M.B., Komanschek B.U. (2000). Dehydration/recrystallization mechanisms, energetics, and kinetics of hydrated calcium silicate minerals: An in situ TGA/DSC and synchrotron radiation SAXS/WAXS study. Chem. Geol..

[23] Biagoni C., Bonaccorsi E., Lezzerini M., Merlino S. (2016). Thermal behaviour of Al-rich tobermorite. Eur. J. Mineral..

[24] Małecki M., Kurdowski W., Walczak P. (2018). Influence of gypsum and limestone, used as mineral additives, on autoclaved aerated concrete properties. ce/papers.

[25] Paradiso P., Santos R.L., Horta R.B., Lopes J., Ferreira P., Colaço R. (2018). Formation of nanocrystalline tobermorite in calcium silicate binders with low C/S ratio. Acta Mater..

[26] Grangeon S., Claret F., Roosz C., Sato T., Gaboreau S., Linard Y. (2016). Structure of nanocrystalline calcium silicate hydrates: Insights from X-ray diffraction, synchrotron X-ray absorption and nuclear magnetic resonance. J. Appl. Crystallogr..

[27] Siauciunas R., Smalakys G., Eisinas A., Prichockiene E. (2022). Synthesis of high crystallinity 1.13 nm tobermorite and xonotlite from natural rocks, their properties and application for heat-resistant products. Materials.

[28] Jansen D., Lothenbach B., Yan Y., Schreiner J. (2023). Synthesis, structural characterization, and thermodynamic properties of 11 Å Al-tobermorite. ce/papers.

[29] Smalakys G., Siauciunas R. (2020). Peculiarities of xonotlite synthesis from the raw materials with different SiO2 activities. J. Therm. Anal. Calorim..

[30] Mao F., Ai H. (2023). A Study on the Hydrothermal Synthesis of Calcium Silicate Products by Calcination of Full-Component Waste Concrete. Sustainability.

[31] Saldia S.Q., Bacosa H., Vegafria M.C., Zoleta J., Hiroyoshi N., Empig E., Calleno C., Cantong W., Ibarra E., Aguilos M. (2023). Combined Potential of Quarry Waste Fines and Eggshells for the Hydrothermal Synthesis of Tobermorite at Varying Cement Content. Preprints.

[32] Diez-Garcia M., Gaitero J.J., Dolado J.S., Aymonier C. (2017). Ultra-Fast Supercritical Hydrothermal Synthesis of Tobermorite under Thermodynamically Metastable Conditions. Angew. Chem. Int. Ed..

[33] Glazev M., Bazhin V. (2021). On the recycling and use of microsilica in the oil industry. E3S Web Conf..

[34] Hou L., Li J.H., Tong L.X. (2012). Preparation and Characterization of Calcium Silicate Slag Based Lightweight Wall Materials. Key Eng. Mater..

[35] Yang Z., Fang C., Jiao Y., Zhang D., Kang D., Wang K. (2023). Study on Crystal Growth of Tobermorite Synthesized by Calcium Silicate Slag and Silica Fume. Materials.

[36] Hurt A.P., Coleman A.A., Ma H., Li Q., Coleman N.J. (2022). Calcium Silicate Hydrate Cation-Exchanger from Paper Recycling Ash and Waste Container Glass. Ceramics.

[37] Rahman H., Li Q., Coleman N.J. (2022). Waste Glass-Derived Tobermorite Carriers for Ag^+^ and Zn^2+^ Ions. J. Compos. Sci..

[38] Wu Y., Pan X., Li Q., Yu H. (2020). Crystallization and phase transition of tobermorite synthesized by hydrothermal reaction from dicalcium silicate. Int. J. Appl. Ceram. Technol..

[39] Mutisya S.M., Miranda C.R. (2018). The surface stability and morphology of tobermorite 11 Å from first principles. Appl. Surf. Sci..

[40] Liao W., Li W., Fang Z., Lu C., Xu Z. (2019). Effect of different aluminum substitution rates on the structure of tobermorite. Materials.

[41] Lothenbach B., Jansen D., Yan Y., Schreiner J. (2022). Solubility and characterization of synthesized 11 Å Al-tobermorite. Cem. Concr. Res..

[42] Siauciunas R., Smalakys G., Dambrauskas T. (2021). Porosity of calcium silicate hydrates synthesized from natural rocks. Materials.

[43] Yan Y., Wang H. (2021). Thermal Behavior and Determination of the Heated Structure of 11Å Anomalous Tobermorite by in situ X-ray Diffraction. Acta Geol. Sin..

[44] Tajuelo Rodriguez E., Garbev K., Merz D., Black L., Richardson I.G. (2017). Thermal stability of C-S-H phases and applicability of Richardson and Groves’ and Richardson C-(A)-S-H(I) models to synthetic C-S-H. Cem. Concr. Res..

[45] Ashraf W., Olek J. (2018). Elucidating the accelerated carbonation products of calcium silicates using multi-technique approach. J. CO2 Util..

[46] Bagheri M., Lothenbach B., Shakoorioskooie M., Scrivener K. (2022). Effect of different ions on dissolution rates of silica and feldspars at high pH. Cem. Concr. Res..

[47] Liu F., Zeng L., Cao J., Li J. (2010). Preparation of ultra-light xonotlite thermal insulation material using carbide slag. Journal of Wuhan University of Technology-Mater. Sci. Ed..

[48] Coleman N.J. (2005). Synthesis, structure and ion exchange properties of 11Å tobermorites from newsprint recycling residue. Mater. Res. Bull..

